# Pennsylvania

**Published:** 1899-09

**Authors:** 


### PENNSYLVANIA STATE MEETING.

The semi-annual meeting of this Association will be held at Scranton on September 19, 1899.

Interesting reports from the several standing committees are announced, especially those of Sanitary Control, Legislation, and Intelligence and Education.

The work of the State Livestock Sanitary Board for the past six months will be reviewed by State Veterinarian Leonard Pearson.

"Some Notes from Practice," a subject of much interest at all times, will be specially true when they emerge from so observing a practitioner as Dr. E. Mayhew Michener, of North Wales.

"The Field of Cattle Pathology" will afford a topic for the paper of Dr. D. C. Stanton, of Factoryville.

"The Ways and Doings of the Œstrus Ovis" will be treated by Dr. J. F. Butterfield, of South Montrose.

"A Veterinarian's Daily Observations" will afford Dr. W. O. Phillips, of Reading, a topic for consideration.

"Reports of Unusual Cases," by Dr. Jacob Helmer, of Scranton, will bring out many interesting points from a busy man's practice.

Dr. F. S. Allen, of Philadelphia, will offer a paper, and several others have promised contributions, subjects to be selected.

The local Committee of Arrangements and the Reception Committee are working hand-in-hand, and we are sure that unless all past signs fail there will be an outpouring of the profession that Scranton may be as equally proud of as any of the other interior cities of the State have entertained.

